# Effects of Grazing on Above- vs. Below-Ground Biomass Allocation of Alpine Grasslands on the Northern Tibetan Plateau

**DOI:** 10.1371/journal.pone.0135173

**Published:** 2015-08-18

**Authors:** Chaoxu Zeng, Jianshuang Wu, Xianzhou Zhang

**Affiliations:** 1 Lhasa Plateau Ecosystem Research Station, Key Laboratory of Ecosystem Network Observation and Modelling, Institute of Geographic Sciences and Natural Resources Research, Chinese Academy of Sciences, Beijing, China; 2 University of Chinese Academy of Sciences, Beijing, China; 3 Functional Biodiversity, Dahlem Center of Plant Sciences, Free University of Berlin, Berlin, Germany; Institute of Tibetan Plateau Research, CHINA

## Abstract

Biomass allocation is an essential concept for understanding above- vs. below-ground functions and for predicting the dynamics of community structure and ecosystem service under ongoing climate change. There is rare available knowledge of grazing effects on biomass allocation in multiple zonal alpine grassland types along climatic gradients across the Northern Tibetan Plateau. We collected the peak above- and below-ground biomass (AGB and BGB) values at 106 pairs of well-matched grazed vs. fenced sites during summers of 2010–2013, of which 33 pairs were subject to meadow, 52 to steppe and 21 to desert-steppe. The aboveground net primary productivity (ANPP) was represented by the peak AGB while the belowground net primary productivity (BNPP) was estimated from ANPP, the ratio of living vs. dead BGB, and the root turnover rate. Two-ways analyses of variance (ANOVA) and paired samples comparisons with *t*-test were applied to examine the effects of pasture managements (PMS, i.e., grazed vs. fenced) and zonal grassland types on both ANPP and BNPP. Allometric and isometric allocation hypotheses were also tested between logarithmically transformed ANPP and BNPP using standardized major axis (SMA) analyses across grazed, fenced and overall sites. In our study, a high community-dependency was observed to support the allometric biomass allocation hypothesis, in association with decreased ANPP and a decreasing-to-increasing BNPP proportions with increasing aridity across the Northern Tibetan Plateau. Grazing vs. fencing seemed to have a trivial effect on ANPP compared to the overwhelming influence of different zonal grassland types. Vegetation links above- and below-ground ecological functions through integrated meta-population adaptive strategies to the increasing severity of habitat conditions. Therefore, more detailed studies on functional diversity are essentially to achieve conservation and sustainability goals under ongoing climatic warming and intensifying human influences.

## Introduction

The allocation of annual biomass production to different structures/organs is a central concept of plant life-history theory and is important to the plant’s growth and survival. In addition to performance and fitness of plant individuals, a better understanding of biomass production, allocation and storage between shoots and roots is also needed for the prediction of macro-ecological dynamics resulting from ongoing climatic change [1]. However, whether a general rule governs biomass allocation along environmental and disturbance gradients and across different ecological levels, from species to ecosystems, remains contentious [2–8].

The Tibetan Plateau is one of the most fascinating regions for scientific research. The alpine grasslands on this plateau are sensitive and vulnerable to both climate change and grazing disturbances [9–11]. Approximately 114,300 km^2^ of grassland, accounting for 17.2% of natural grassland in the Tibet (Xizang) Autonomous Region, has been degraded due to historical overgrazing, irrational land use and climatic warming since the 1980s [12, 13]. Air temperature on the plateau has increased by 0.3°C per decade since the 1960s, which is three times the global average [9, 14]. Because grasslands on the Tibetan Plateau play an important ecological role in protecting the headwaters of Asia’s major rivers, such as the Yellow, Yangtze and Lantsang-Mekong Rivers, grassland degradation threatens the livelihood of residents in the local and surrounding regions [9, 14]. Since 2004, the Chinese government has launched a series of ecological restoration projects and conservation policies, such as the Livestock Grazing Exclusion & Rangeland Fence Construction, the Sustainable Control of *Lagomorphs* (*Ochotona*) on Damaged Grassland, and Providing Allowances and Awards to Local Herdsmen Families [15–17], to promote degraded pasture recovery and to balance the livestock rate with forage productivity. Therefore, fenced vs. grazed pastures across zonal grassland types provide us with a natural comparative experiment to test the effects of livestock grazing on the basic functionalities of alpine grasslands along environmental gradients across the Northern Tibetan Plateau.

Recently, ecologists have contributed to a better understanding of plant biomass allocation on the Tibetan Plateau, across ecological levels, from plant individuals [18, 19] and functional groups/life forms [19, 20] to zonal grassland types [21, 22]. However, controversial findings further stimulate the debate on plant biomass allocation between the shoots and roots of alpine grassland plants on this plateau. For example, both isometric [21] and allometric biomass allocation hypotheses [22, 23] have been reported for alpine steppes across the Northern Tibetan Plateau. However, an allometric relationship is not a general rule governing biomass allocation of herbaceous species at the plant individual level [18, 19] and the taxonomic group level [20] on Tibetan Plateau; habitat climate vary from alpine humid, semi-arid, to extreme arid and zonal grassland types from meadows, steppes to desert-steppes. Ma et al. [19] found that, for the perennial herbaceous plants, the reproductive outputs decrease, fine roots increase and leaf fractions remain constant along an increasing elevation gradient in the Central Tibetan Plateau. Wu et al. [20] emphasized that different functional groups of sedges, legumes, grasses and forbs specifically respond to a westward increasing aridity gradient across the Northern Tibetan Plateau. Furthermore, Yang, Fang [21] collected biomass data on freely grazed pastures during the period 2001–2005 when no enclosures were present; thus, the aboveground biomass might be underestimated due to livestock grazing. In contrast, Wu et al. [22] and Wu et al. [20] conducted field surveys on multiple pastures protected with metal fences. However, no studies have rigorously examined the effects of livestock grazing on biomass allocation compared to adjacent pastures that have been fenced for several years across the three most zonal alpine grassland types on the Northern Tibetan Plateau.

Therefore, we need an experimental design that includes grazed vs. fenced well-matched sites to determine whether the fences efficiently promote degraded pasture recovery and whether grazing alter biomass allocation patterns of alpine grasslands at the community level on this plateau. Concretely, in this study we particularly aim (1) to explore whether a significant difference exists between above- and below-ground net primary productivity (ANPP and BNPP) in alpine meadows, steppes and desert-steppes; (2) to examine whether the fences have resulted a significant increase or decrease in the productivity/biomass allocated to above- and below-ground components, compared with neighboring grazed sites; and finally, (3) to clarify which biomass allocation hypothesis, allometric or isometric, is supported by our data from both the grazed vs. fenced sites within and across zonal grassland types.

## Materials and Methods

No specific permits were required for the samples collected from any of the sites, and the field studies did not involve endangered or protected species.

### Study area

The Northern Tibetan Plateau (29°53′–36°32′ N; 78°41′–92°16′ E; 597, 000 km^2^) is located in the northwestern hinterlands of the Qinghai-Tibetan Plateau. In May 2009, we established an alpine grassland transect of multiple paired grazed vs. fenced sites across the Northern Tibetan Plateau for a long-term large-scale ecological research [24, 25]. The herbaceous plants in this region generally sprout in early May, reach their peak coverage in mid-August, and finish reproduction before late September. Along this transect, growing season temperature (GST) increases from 4.8°C in the most eastern sites in Amdo to 11.6°C in the most western ones in Rutog, whereas growing season precipitation (GSP) decreases from 470 mm to 30 mm [25]. From east to west, the transect traverses three zonal alpine grassland types: alpine meadow (AM) dom inated by *Kobresia pygmaea*, alpine steppe (AS) dominated by *Stipa purpurea*, and alpine desert-steppe (ADS) co-dominated by *S*. *purpurea* and *S*. *glareosa*. The AM, the highest productivity type, occupies relative richer soils where the mean annual precipitation (MAP) is greater than 450 mm; the AS (intermediate productivity) is widespread on soils with moderate nutrient availability where the MAP is greater than 250 mm but less than 450 mm; and the ADS, with the lowest productivity type, is found on poor and arid soils where MAP is less than 250 mm (Table 1) [20, 24, 26]. Limited by many reasons (time, harsh environment, inconvenient traffic) on the vast Northern Tibetan Plateau, we could not obtain enough accurate information (timing, intensity, and frequency) to describe the grazing activities that occurred in the open pastures in each years. Instead, we sought these information to local government officials in summer 2012; they reported that one sheep unit needs approximately 1.0–2.0 hm^2^ in AM, 3.0–4.0 hm^2^ in AS and 6.0–8.0 hm^2^ in ADS per year, respectively [27]. In general, sheep unit has been used to evaluate the pasture supporting capacity, such as, yak, will be converted into standardized sheep unit by one yak equivalent five sheep[28].

**Table 1:** Geographic, climatic and vegetation information for well-matched grazed vs. fenced sites across the Northern Tibetan Plateau (NTP).

AGTs	Number	Longitude (°E)	Latitude (°N)	Altitude (m)	GSP (mm)	AccT (°C)	GSP/AccT (mm °C^-1^)	SR	Grazing Intensity (one sheep / n hm^2^ pasture)
AM	33	91.4815–91.9080	31.5934–32.3035	4531–4730	394.3–449.1	1092.0–1251.0	0.31–0.40	23.30 (14–36)	1.0–2.0
AS	52	85.0797–91.0217	31.3641–33.2069	4541–4995	267.9–380.3	807.5–1515.5	0.19–0.25	11.80 (5–21)	3.0–4.0
ADS	21	81.8218–84.2025	32.0804–33.1733	4440–4671	135.1–231.1	1567.4–1720.8	0.08–0.15	6.70 (3–10)	6.0–8.0
NTP	106	81.8218–91.9080	31.3641–33.2069	4440–4995	135.1–449.1	807.5–1771.5	0.08–0.40	13.62 (3–36)	1.0–8.0

AGTs = Alpine grassland types, AM = alpine meadow, AS = alpine steppe and ADS = alpine desert-steppe

GSP = growing season precipitation; AccT = accumulated active temperature ≥ 5°C; GSP/Acct = an alternative moisture index; SR = species richness at each site.

Climatic variables are from Wu, Shen [26],SR are from Wu, Zhang [25] and grazing intensities are from Wu, Zhang [27].

### Biomass collection

In this study, we measured the peak above- and below-ground biomass (AGB and BGB) during the summers of 2010–2013 at well-matched grazed vs. fenced sites, of which 33 pairs were meadow, 52 were steppe and 21 were desert-steppe (Table 1). The effects of large domestic herbivores on biomass accumulation are difficult to assess; therefore, the grazed sites in this study are strictly limited to the winter pastures that are only grazed during cold months but not grazed before field sampling in August [29]. Thus, the peak AGB reasonably approximates the ANPP [26] and can be used to estimate the BNPP from biomass samples. Most of fenced pastures have been excluded from domestic large herbivores since summer 2006 or spring 2007. Open grazed pastures within a distance of two kilometers from the enclosure boundaries were chosen as pairs to the fenced sites, and the slope, aspect, soils and climatic conditions were considered to ensure that each pair of grazed and fenced sites were as similar as possible [29].

The standing plant biomass aboveground and the dead-and-living mixed roots were sampled on five 0.25-m^2^ quadrats. These quadrates were systemically located along a random 100-m transect line at 20-m intervals within a randomly selected plot of 200 m × 200 m in size. The root biomass in the uppermost 20-cm-thick soil layer was reported by Li, Zhang [24] to account for 74.85%–87.29% of the total BGB in this region. Limited by budgets, time and the harsh environments, we could not excavate many soil cores so would likely have destroyed the sensitive and vulnerable alpine vegetation. Alternatively, we sampled roots from three of the five quadrats at each plot. In 2010 and 2011 we sampled five to nine soil cores of 0.05 m (diameter) × 0.20 m (depth), whereas in 2012 and 2013 we excavated one soil block of 0.25 m (length) × 0.25 m (width) × 0.20 m (depth) for roots. To compare these samples, the BGB was transformed into dry mass per square meters (g m^-2^) to a depth of 0.20 m. Finally, the soil samples were fully soaked, cleaned under running water to remove soil particles, and sieved over a 0.2 mm mesh to separate the roots. All the AGB and BGB samples were oven-dried at 65°C for 48 h and weighed.

### BNPP estimation

We followed the algorithm from Gill, Kelly [30] to estimate the BNPP:

$$\text{BNPP}=\text{BGB}\times \frac{Live\;BGB}{BGB}\times turnover$$ (1)

where: BGB is the belowground biomass in g m^-2^ and is equal to the sum of dead and living roots; In this study, mean (live BGB)/BGB was 0.72 for the AM cited from Zhou [31], and 0.79 for AS and ADS according to Wu, Shen [20]. The turnover rate is the proportion of roots that are produced or died annually [32, 33]. To date, no references are available for root turnover rates on the Northern Tibetan Plateau. Therefore, we related turnover to the ANPP, as suggested by Gill, Kelly [30] (S1 Table):

$$\text{turnover}=0.0009\;\text{g}\cdot m^{-2}\left(\text{ANPP}\right)+0.25\;yr^{-1}$$ (2)

### Statistical analysis

Ratios, which include both a numerator and denominator, have been criticized by Müller, Schmid [34] because they can be changed by either the numerator, the denominator, or both. The R:S ratio is important for modelling carbon-cycling in various terrestrial ecosystems [35]; however, this ratio has been criticized for ignoring the differential functionalities between stem and leaf aboveground, and between fine and coarse roots belowground [36–38]. Therefore, as Poorter, Niklas [8] suggested, the biomass allocation was explored as productivity fraction from the perspective of bivariate allometric analyses. In addition to statistical descriptions, a two-way analysis of variance (ANOVA) was applied to disentangle the effects of the community types (AM, AS, and ADS) and pasture managements (grazed vs. fenced here) on both the ANPP and BNPP across the Northern Tibetan Plateau. The ANPP and BNPP were firstly logarithmically transformed, and then the allometric slope and intercept were determined via a standardized major axis test using SMATR 2.0 [39]. All the statistical analyses were performed using the R 3.1.1 software package [40], and the figures were plotted in Sigma Plot 12.5 (Systat Software Inc., Chicago, IL, USA).

## Results

### Variations in ANPP and estimated BNPP

**Fig 1:**
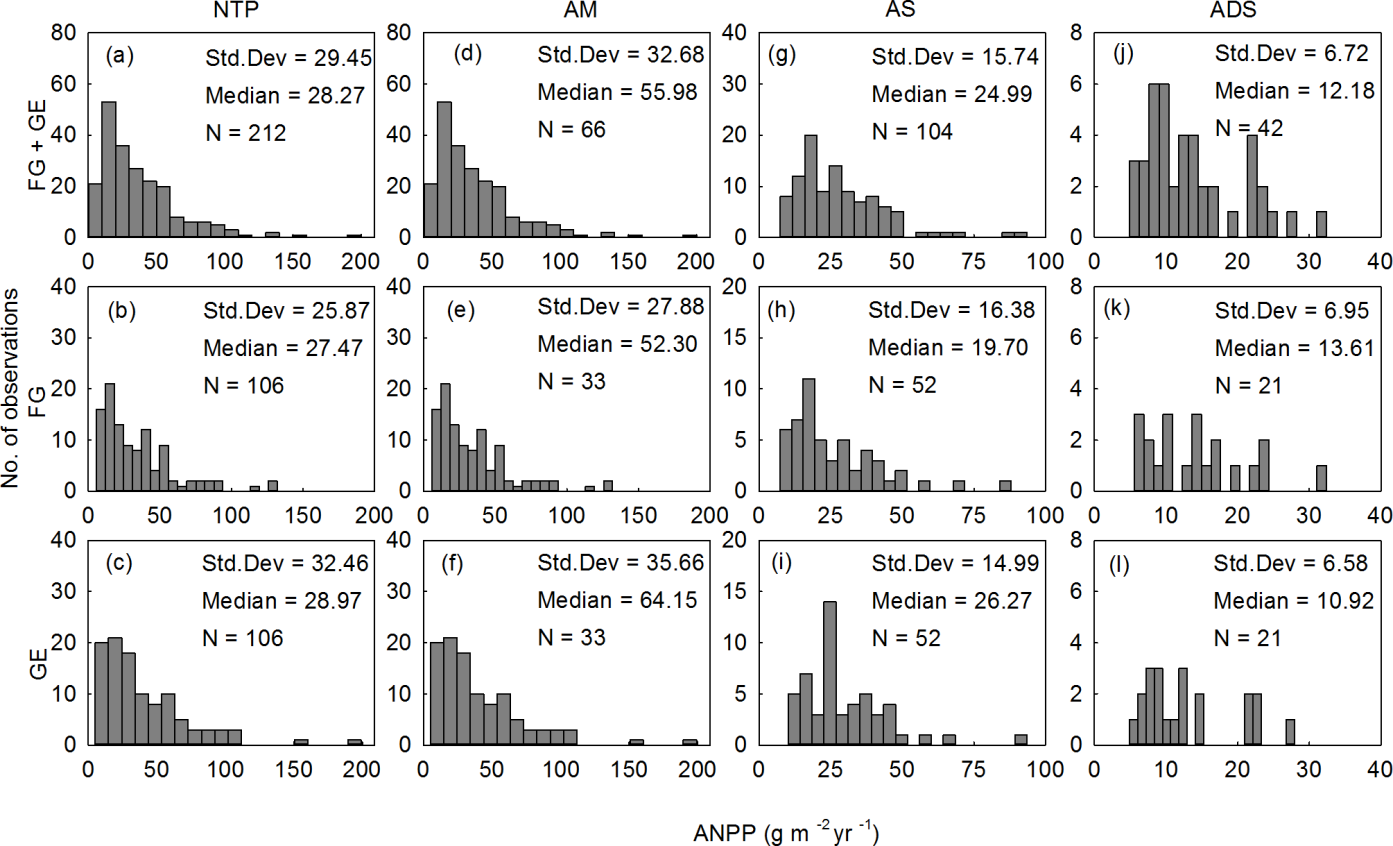
Frequency distributions of aboveground net primary productivity (ANPP) of alpine grasslands on the Northern Tibetan Plateau (NTP). ANPP is equal to the peak aboveground biomass (AGB). AM = alpine meadow; AS = alpine steppe; ADS = alpine desert-steppe. FG = free grazed; GE = grazed excluded.

**Fig 2:**
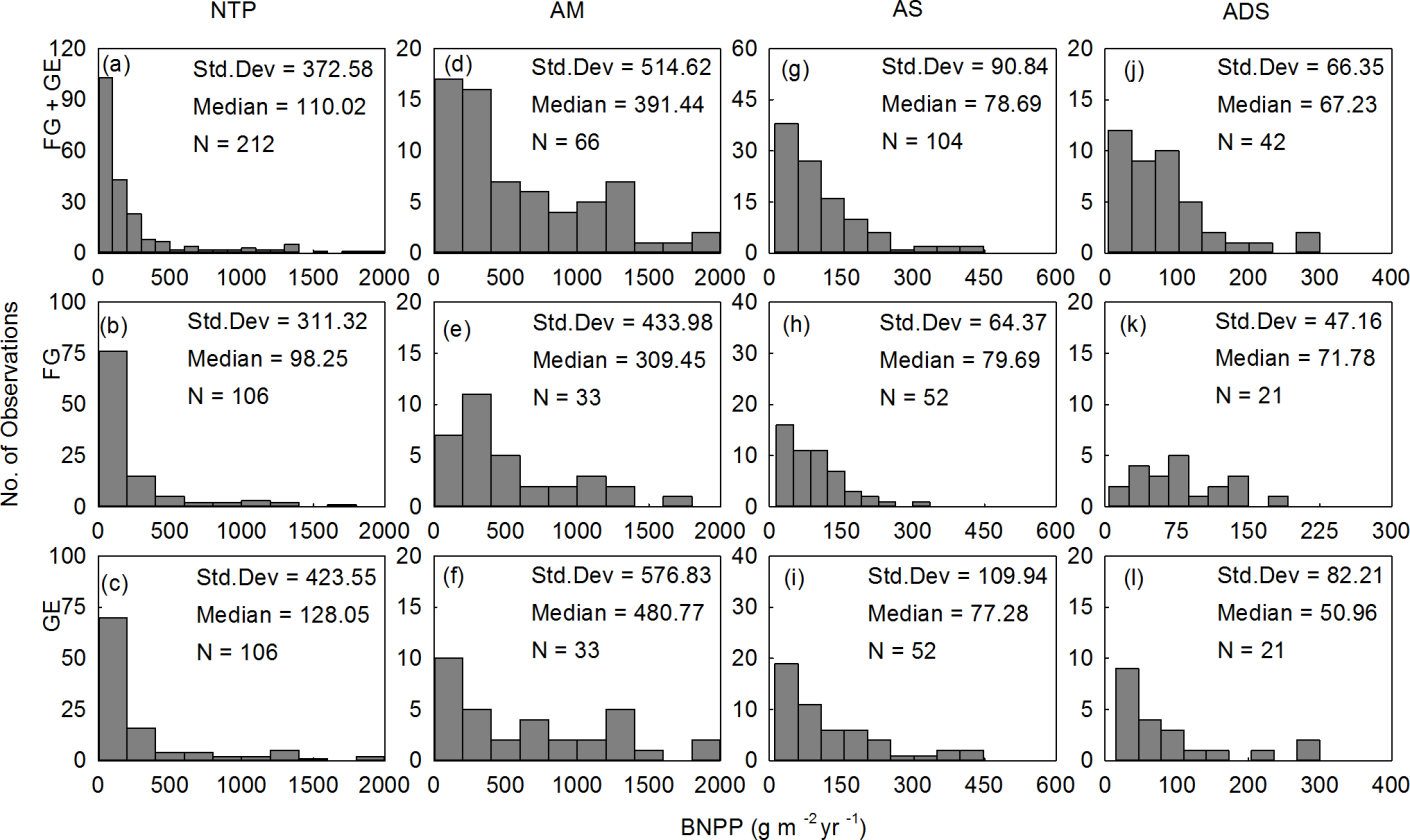
Frequency distributions of belowground net primary productivity (BNPP) of alpine grasslands on the Northern Tibetan Plateau (NTP). BNPP was estimated from ANPP and the turnover rate of belowground biomass (BGB). AM = alpine meadow; AS = alpine steppe; ADS = alpine desert-steppe. FG = free grazed; GE = grazed excluded.

Considerable variations in both the ANPP and estimated BNPP were found across the Northern Tibetan Plateau (Figs 1 and 2). The ANPP ranged from 4.86 to 199.10 g m^-2^ yr^-1^, and the BNPP ranged from 4.12 to 1991.29 g m^-2^ yr^-1^ (Table 2). When the grazed and fenced sites were pooled together, the ANPP vs. BNPP medians were 55.98 vs. 391.44 g m^-2^ yr^-1^ for meadows, 24.99 vs. 78.69 g m^-2^ yr^-1^ for steppes, and 12.18 vs. 67.23 g m^-2^ yr^-1^ for desert-steppes (Table 2). The mean ANPP values under grazed vs. fenced management in the alpine meadow and steppe zones were approximately 4.1 vs. 5.6 and 1.9 vs. 2.1 times higher, respectively, than the values of the alpine desert-steppe zone. Consistently, BNPP values under grazed vs. fenced management in the alpine meadow were approximately 5.4 vs. 5.6 and 6.5 vs. 8.0 times higher for alpine steppe and alpine desert steppe, respectively.

**Table 2:** Statistics of above- and below-ground net primary productivity (ANPP and BNPP) at free grazed (FG), grazed excluded (GE) and pooled (FG + GE) sites on the Northern Tibetan Plateau (NTP).

			ANPP					BNPP				
AGTs	No.	Managements	Maximum	Minimum	Median	Mean	Std. Error	Maximum	Minimum	Median	Mean	Std. Error
AM	33	FG	131.92	21.03	52.30	58.34	4.85	1733.21	24.69	309.45	502.42	75.55
	33	GE	199.10	18.57	64.15	73.58	6.21	1991.29	27.87	480.77	683.35	100.41
	66	FG + GE	199.10	18.57	55.98	65.96	4.02	1991.29	24.69	391.44	592.88	63.35
AS	52	FG	87.92	7.28	19.70	26.43	2.27	335.30	14.33	79.69	93.30	8.93
	52	GE	93.46	10.12	26.27	30.16	2.08	447.68	11.12	77.28	122.44	15.25
	104	FG + GE	93.46	7.28	24.99	28.29	1.54	447.68	11.12	78.69	107.87	8.91
ADS	21	FG	32.44	5.54	13.61	14.22	1.52	191.76	4.12	71.78	77.32	10.29
	21	GE	27.94	4.86	10.92	12.97	1.44	299.42	14.85	50.96	86.26	17.94
	42	FG + GE	32.44	4.86	12.18	13.59	1.04	299.42	4.12	67.23	81.79	10.24
NTP	106	FG	131.92	5.54	27.47	33.94	2.51	1733.21	4.12	98.25	217.50	30.24
	106	GE	199.10	4.86	28.97	40.27	3.15	1991.29	11.12	128.05	289.89	41.14
	212	FG + GE	199.10	4.86	28.27	37.11	2.02	1991.29	4.12	110.02	253.70	25.59

AM = alpine meadows, AS = steppes, ADS = desert-steppes

### Effects of enclosures on ANPP and BNPP

**Fig 3:**
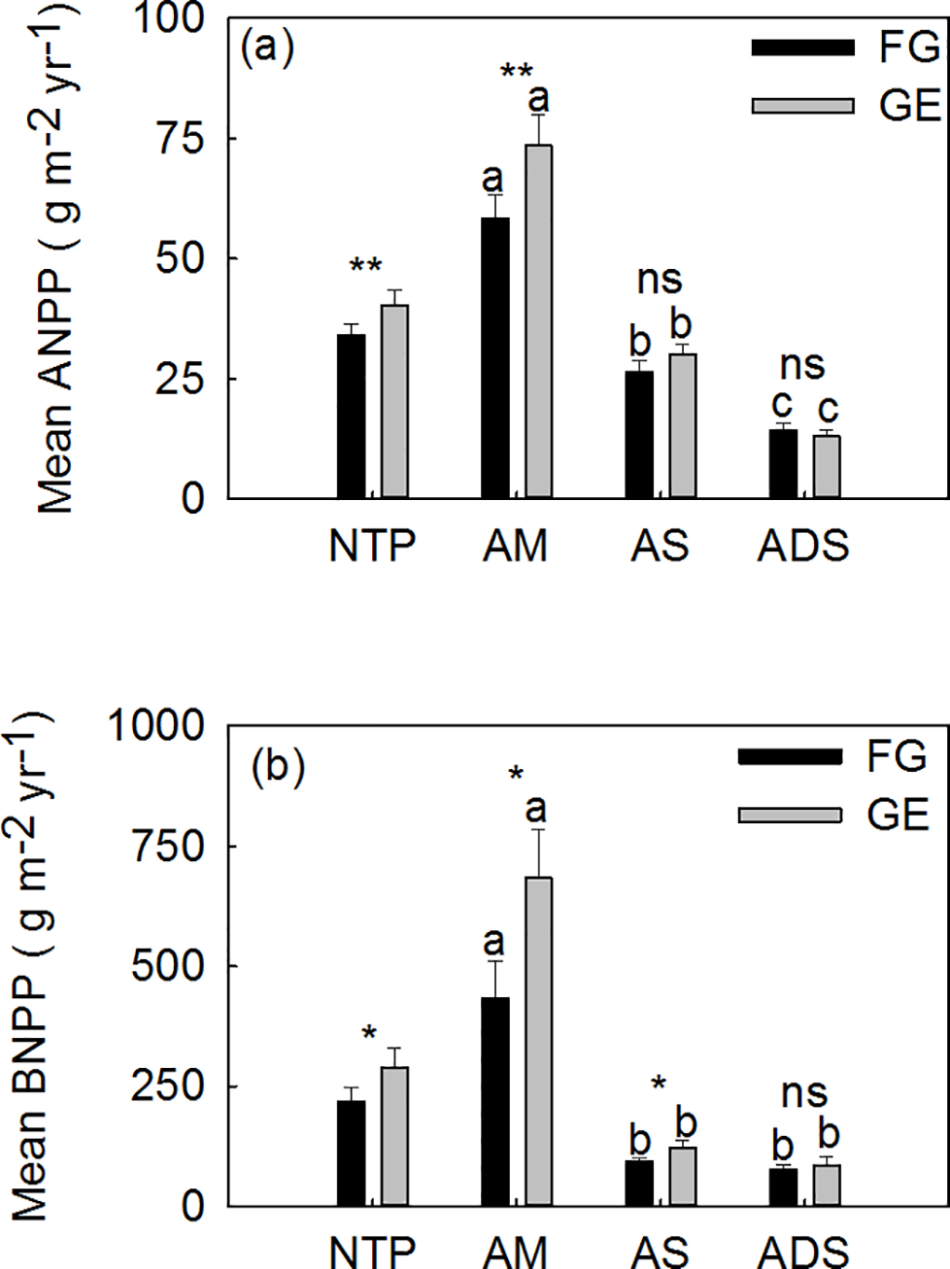
Multiple comparisons of the mean values of above- and below-ground net primary productivity (ANPP and BNPP). A two-way analysis of ANOVA with Turkey’s test was applied to free grazed (FG) and grazed excluded (GE) sites, to determine if ANPP (or BNPP) is significantly different among the three zonal alpine grassland types (AGTs) on the Northern Tibetan Plateau, AM = alpine meadow; AS = alpine steppe; ADS = alpine desert-steppe. Different letters indicate statistically significant differences at *P* < 0.05. Paired samples *t*-tests were conducted to determine if grazing exclusion altered the ANPP and BNPP relative to the adjacent open pastures under livestock grazing. ** and * indicate significant differences at *P* < 0.01 and *P* < 0.05, respectively. The ns means no significance in statistics.

The alpine grassland types (AGTs) had a significant influence on ANPP and BNPP across the Northern Tibetan Plateau (two-way ANOVA, *P* < 0.01, Table 3). The pastures management systems (PMS, grazed vs. fenced) had a significant influence on ANPP (two-way ANOVA, P < 0.05, Table 3) but none significant influenced BNPP. At a regional scale, the exclusion of grazing appeared to increase the ANPP by 6.33 g m^-2^ yr^-1^ and the BNPP by 72.69 g m^-2^ yr^-1^ (Table 2; Fig 3) compared with grazed pastures. However, at the grassland type level, only the ANPP of alpine meadows (*P* < 0.01) and the BNPP of alpine meadow and alpine steppes (*P* < 0.05) were found to be significantly higher in fenced areas (grazed vs. fenced paired samples *t*-test, Fig 3), and neither the ANPP nor BNPP values were significantly affected by grazing exclusion in the alpine desert-steppes.

**Table 3:** Summary of two-way analyses of variance with general linear models of alpine grassland types (AGTs) and pasture management systems (PMS).

Source	*d*.*f*.	ANPP			BNPP		
		M.S.	*F-*value	*P-*value	M.S.	*F*-value	*P*-value
AGT	1	79439	166.049	0.000	8325616	84.648	0.000
PMS	1	2123	4.437	0.036	277760	2.824	0.094
AGT*PMS	1	1950	4.076	0.045	229397	2.332	0.128
Residuals	208	478			98356		

AGTs (meadow, steppe, desert-steppe) were as fixed factors, while PMS (grazed vs. fenced) as random factors, and the interaction of both for above- and below-ground net primary productivity (ANPP and BNPP) on the Northern Tibetan Plateau.

Degrees of freedom (*d*.*f*.), mean squares (M.S.), variance ratio (*F*-value) and significance level (*P*-value) are shown.

### Allometric log ANPP- log BNPP relationship

**Fig 4:**
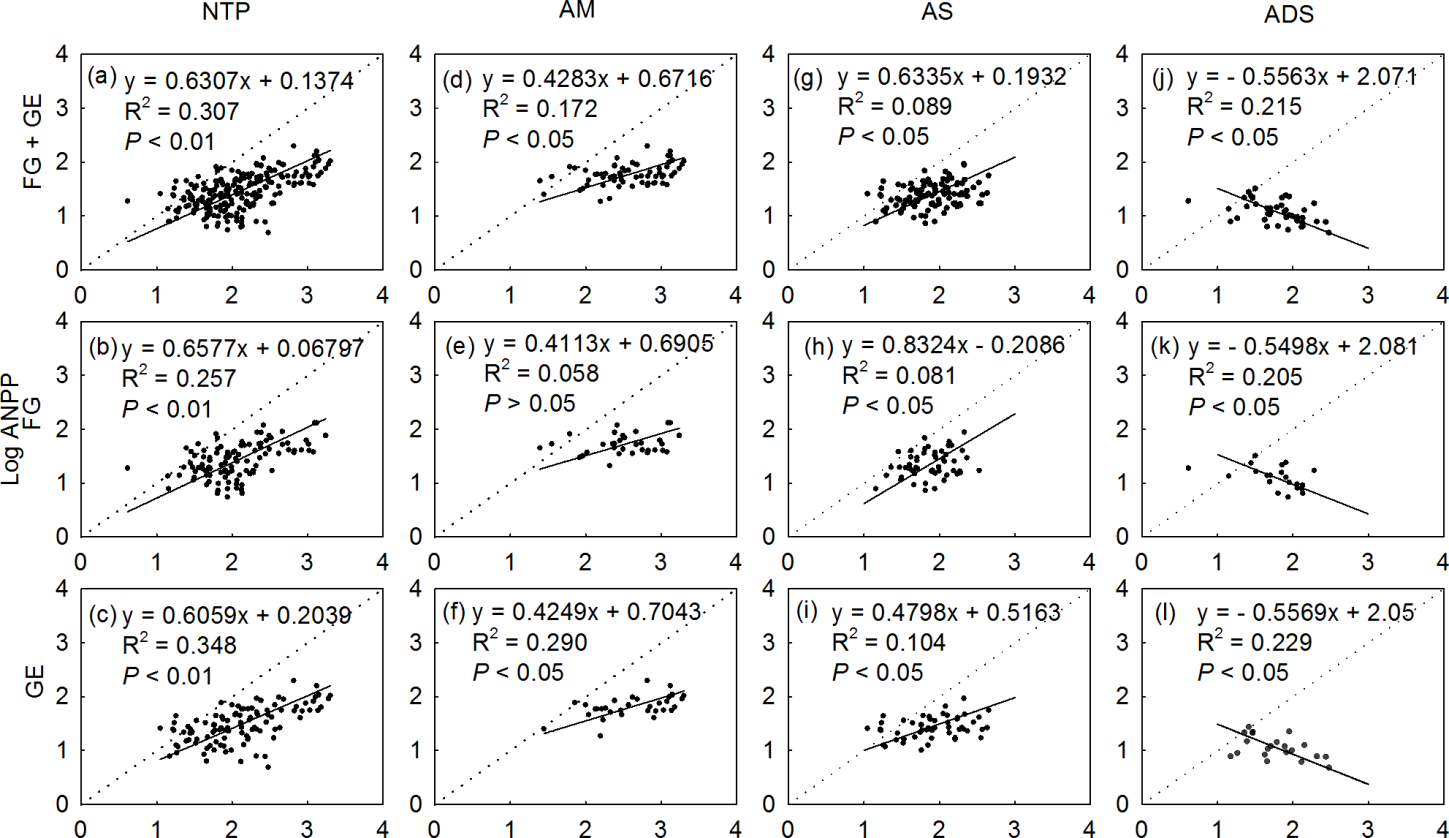
Standardized major axis analyses (SMA) of relationships between Log (ANPP) and Log (BNPP) at grazed sites, fenced sites, and pooled sites within and across zonal alpine grassland types on the Northern Tibetan Plateau (NTP). AM = alpine meadow; AS = alpine steppe; ADS = alpine desert-steppe. FG = free grazed; GE = grazed excluded.

From the bivariate allometric analyses, we estimated slopes (α) and y-intercept (Log β) across the entire Northern Tibetan Plateau and within each alpine grassland type for pastures under grazed and fenced pastures (Table 4; Fig 4). We found that the biomass allocation between Log ANPP and Log BNPP supported the allometric hypothesis, except for the isometric relationship in grazed pastures in alpine steppe zone, with α ranging from 0.636 to 1.089 (*P* = 0.180).

**Table 4:** Results of standardized major axis (SMA) analyses between Log ANPP and Log BNPP on the Northern Tibetan Plateau (NTP).

AGTs	PMS	n	αα	95% of CI	Log ββ β	95% of CI	*F*	*p*
NTP	FG + GE	212	0.631	0.563–0.706	0.137	-0.017–0.292	69.009	0.000
	FG	106	0.658	0.557–0.777	0.068	-0.166–0.302	26.043	0.000
	GE	106	0.606	0.518–0.708	0.204	-0.005–0.413	43.518	0.000
AM	FG + GE	66	0.428	0.342–0.537	0.672	0.416–0.928	70.213	0.000
	FG	33	0.411	0.290–0.583	0.691	0.314–1.067	33.548	0.000
	GE	33	0.425	0.314–0.576	0.704	0.352–1.056	40.597	0.000
AS	FG + GE	104	0.634	0.526–0.763	0.193	-0.038–0.424	24.995	0.000
	FG	52	0.832	0.636–1.089	-0.209	-0.641–0.224	1.852	0.180
	GE	52	0.480	0.368–0.626	0.516	0.261–0.771	35.899	0.000
ADS	FG + GE	42	-0.556	-0.736 –-0.421	2.071	1.783–2.358	19.621	0.000
	FG	21	-0.550	-0.833 –-0.363	2.081	1.651–2.511	9.624	0.006
	GE	21	-0.557	-0.839 –-0.370	2.050	1.622–2.478	9.459	0.006

The slopes (α) and y-intercepts (Log β) are from SMA analyses of the Log ANPP–Log BNPP relationships on the NTP for free grazed (FG), grazed excluded (GE) pastures and pooled (FG + GE) within and across the alpine meadow (AM), steppe (AS) and desert-steppe (ADS) zones. For α and Log β, 95% of confidence intervals (CI) are provided. Null Hypothesis is α = 1.0.

## Discussion

In the present study, the productivity of alpine grasslands was also found community-dependent across the Northern Tibetan Plateau. The ANPP and BNPP values of the communities exhibit the same pattern: meadow (65.96 ± 4.02 g m^-2^) > steppe (28.29 ± 1.54 g m^-2^) > desert-steppes (13.59 ± 1.04 g m^-2^) for ANPP, and meadow (592.88 ± 63.35 g m^-2^) > steppe (107.87 ± 8.91 g m^-2^) > desert-steppes (81.79 ± 10.24 g m^-2^) for BNPP (Table 2). Our results are consistent with the significant decreasing trends in AGB and BGB associated with the westwardly decreasing precipitation gradient in this region [24, 26]. The average root biomass density was reported by Li, Zhang [24] to feature the following order: meadows (3,799.14 ± 1,311.62 g m^-2^) > steppes (1,412.77 ± 200.75 g m^-2^) > desert-steppes 919.07 ± 321.93 g m^-2^). These root mass density values are approximately 6.4, 13, and 11 higher than our BNPP estimates for the corresponding grassland types. In our study, the BNPP:ANPP ratio can be calculated as meadow (9.0), steppe (3.8), desert-steppe (6.01), which are larger than the R:S values reported for alpine meadows (6.8) and smaller than steppes (5.2) by Yang, Fang [21] and smaller than steppes (11.83) reported by Wu, Hong [22] at the community level. Accurately estimating the R:S value for alpine grasslands is challenging because dead roots are generally mixed with live ones and are difficult to identify. Since Gill, Kelly [30] developed an algorithm for BNPP estimation in grasslands, increasing numbers of studies [22, 41, 42] have estimated the BNPP based on the AGB, living vs. dead BGB, and root turnover.

Scurlock, Johnson [43] reported that the proportion of BNPP accounting for the total NPP ranges from 40% in savannas to 88% in cold steppes. For temperate grasslands in China, the BNPP proportion varies from 50% to 66% [44] while in alpine meadows of Qinghai Province, the values range from 53% to 68% [45]. We estimated our BNPP proportions were estimated as 90% for meadows, 79% for steppes, and 86% for desert-steppes across the Northern Tibetan Plateau. These BNPP proportions are larger than both temperate grasslands in China and previously studied alpine meadows on the Tibetan Plateau; however, the values are consistent with high elevation herbs, which prefer to suppress aboveground stem, proportionally increase fine root components, and invest more biomass into persistent storage structures [37, 46].

Compared to previous allometric analyses of biomass allocation across multiple sites on the vast Tibetan Plateau, we overserved an evident pattern that more biomass, in term of productivity, being allocated to belowground components at the community level in association with decreasing precipitation and increasing temperature gradients across zonal grasslands in this region (Tables 1 and 2). In terms of optimal allocation, plants generally allocate more biomass to structure/function that is most limited in their habitat [34, 47–49]. For example, plants in arid and nutrient-poor ecosystems allocate more biomass to roots to better access water and nutrients in deeper soils than those in humid and nutrient-rich environments. In addition to the westwardly increasing aridity (Table 1), soil nutrients also decreases from meadow, to steppe and then to deserts [24, 50]. Therefore, plants must invest more biomass into belowground organs for more efficiently uptake of water and nutrients from deeper soils under more severe habitat conditions. In fact, Wu, Shen [20] have reported that several plant taxonomic groups–grasses, sedges, legumes, and forbs–have specific strategies for biomass allocation among the functional components of leaves, stems, roots and reproductive outputs, allowing them to adapt to the aridity gradient across the Northern Tibetan Plateau. Therefore, the visibly different biomass allocation patterns among the three zonal alpine grassland types are likely related to community assemblage comprising different functional groups. Climate variables are increasingly accept as the main factors responsible for spatial and/or temporal variations in species richness, diversity indices, productivity, as well as relationships in alpine grasslands across the Tibetan Plateau [21, 24–26, 51–54]. However, in addition to climatic controls, grazing disturbances are an important exterior regulator for plant performance, community composition and vegetation dynamics in grasslands [55–58]. Therefore, improving our understanding of the biomass allocation responses to grazing disturbances is essential in light of ongoing climatic warming. Over a short distance, climatic conditions can be viewed as homogeneous between paired grazed vs. fenced sites in our study; therefore, we can reasonably examine whether fences significantly alter biomass allocation relative to the adjacent grazed pastures.

Grazing may alter community assemblage, thereby affecting plant allocation pattern at the community level [59]. However, there is continuous debate regarding how grazing affect root biomass accumulation and belowground productivity; positive [60, 61], negative [62, 63] and even no effect [64], have been reported in grasslands all over the world. In our study, the slopes of the allometric relationship between Log ANPP and Log BNPP are considerably different among zonal grassland types. When the data from grazed and fenced sites are pooled together, the following pattern emerges: steppe (0.53–0.76) > meadow (0.34–0.54) > desert-steppes (-0.74 –-0.42) (Table 4); however, no significantly changes exist between grazed and fenced sites within a given grassland type. Our results further confirmed the allometric and community-dependent biomass allocation patterns (Fig 4; Table 4), and differ from previous reports that have described allometric analyses of biomass allocation between above- and below-ground biomass in Tibetan alpine grasslands. For example, Yang, Fang [21] reported isometric relationships between Log AGB and Log BGB at the community level and found no significant difference in the isometric slopes between alpine meadow (0.82–1.02) and steppe sites (0.58–1.01) within the 95% confidence intervals. In contract, Wu, Hong [22] also observed an allometric biomass allocation pattern, with the slope ranging from 0.75 to 1.01, in alpine steppes in the same region as our study. Compared with adjacent fenced sites, grazing has slightly decreased both ANPP and BNPP (Table 2); however, the effect of grazing vs. fencing is trivial compared with the overwhelming influence of the alpine grassland types on aboveground components (Table 3). From our results, we observed that neither meadows nor desert-steppes exhibit a significant difference in the allometric slopes between grazed and fenced sites on the Northern Tibetan Plateau. We even found that the slope of allometric analyses for grazed steppes (0.64–1.09) were consistent with the slope of 0.58–1.01 reported by Yang, Fang [21]. The slope of 0.37–0.63 for fenced steppes is more soundly support by the allometric allocation hypothesis rather than the isometric allocation hypothesis.

In addition to the habitat properties and grazing disturbances, we commended that interior vegetation regulators, such as local species pool, community assembly and plant functional diversity, should be also considered to understand the differential patterns of both biomass allocation and productivity partitioning across zonal alpine grasslands. No general pattern of species richness-productivity relationship, after removing influences of climatic and edaphic variables, was reported by Ma, He [65] at a large regional scale or by Wang, Luo [66] along an altitude gradient. In these studies, the parallel responses of both diversity and productivity to environment variables are overstated. In contrast, Wu, Shen [26] proved that the community assembly, i.e., functional group composition, is as important as climatic variables in shaping the spatial productivity pattern across the Northern Tibetan Plateau. Wu, Zhang [27] further confirmed that taxonomic plant groups–grasses, sedges, legumes, and forbs–respond to prolonged grazing exclusion in different ways: legumes benefited less than other groups, whereas grasses benefited most from livestock exclusion. Therefore, a certain degree of complementarity may exist among these different taxonomic groups in alpine grassland communities and likely regulates the biomass allocation at the community level. For example, alpine meadows with more complicated species composition, where plants can be recruited from a relative larger local species pool (16–36), and likely result in an apparent consistency of biomass allocation pattern between grazed and fenced sites. Due to the long-term natural selection and co-evolution between alpine plants, arid climate and poor soils, the local species pool is very small in the alpine desert-steppe zone, and only 4–11 species can be identified within an area of approximately four hectares, as reported by Wu, Shen [26]. Plants in alpine desert-steppes belonging to different taxonomic groups have a similarly large proportion of root biomass [20] to survive under harsher physical (drought) stresses. The arid climate and poor soils are likely responsible for the negative slopes in alpine desert-steppe zone, where plants must invest more biomass and nutrients to roots and meristems. Therefore, the negative slopes for alpine desert-steppes are consistent with larger R:S ratios in drier environments, as reported in previous studies all over the world [67]. No evident relationship between species richness and productivity in either alpine meadow or desert-steppe has been reported, but a positive linear relationship was found for alpine steppes in Wu, Shen [26]. Therefore, the mechanisms of the species pool are likely different: the larger species pool in humid alpine meadows allows for community productivity to be more resistant to grazing disturbance, whereas the smaller species pool in arid desert-steppes zone appears to exhibit no significant differences in community biomass allocation between grazed and fenced sites due to the high uniformity in the species’ adaptive strategies to harsh drought stress [20, 26]. Furthermore, short-term grazing exclusion may not result a significant variation in the number of species belonging to different taxonomic groups at the local scale and will likely only alter the relative abundance of individual species in the greater community [29]. As grazing exclusion expands, considerable variability in productivity will develop among different taxonomic groups, as mentioned above [27], which is likely due to the groups’ differential adaptive strategies for biomass partitioning [20] and functional traits related to water usage [50].

## Conclusions

Vegetation links above- and below-ground ecological functions thought integrated meta-population strategies for biomass allocation between roots and shoots. Our results support the allometric biomass allocation hypothesis, rather than the isometric allocation one. A high community-dependency was found for biomass allocation, such that the ANPP decreases and the BNPP proportion shows a decreasing-to-increasing pattern with increasing aridity across the Northern Tibetan Plateau. Grazing vs. fencing appeared to have a trivial effect on the ANPP compared with the overwhelming influence of different zonal grassland types, due to the long-term natural selection and co-evolutionary process between alpine plants and the alpine-arid climate on this plateau. Therefore, we conclude that the basic functionality of Tibetan alpine grasslands is closely related to the community composition of functional/taxonomic groups with differential adaptive strategies to the severe habitat conditions. Therefore, more detailed studies on functional diversity are essential to achieve conservation and sustainability goals with ongoing climatic warming and intensifying human influences.

## Supporting Information

S1 Table Above-ground net primary productivity (ANPP g m^-2^ yr^-1^) and root turnover rates (yr^-1^), which were calculated by Gill’s algorithm for alpine meadow (AM), alpine steppe (AS) and alpine desert steppe (ADS) under grazing (FG) and grazing exclusion (GE) in Table A, B and C, respectively. (DOCX)
