# Supplementary material for: Effects of Grazing on Above- vs. Below-Ground Biomass Allocation of Alpine Grasslands on the Northern Tibetan Plateau
Source: PLoS One. 2015 Aug 18;10(8):e0135173. doi: 10.1371/journal.pone.0135173 (PMC4540449; doi:10.1371/journal.pone.0135173)
Supplement: S1 Table — (DOCX) [file pone.0135173.s001.docx]

**S1 Table A. Above-ground net primary productivity (ANPP g m^-2^ yr^-1^) and root turnover rates (yr^-1^), which were calculated by Gill’s algorithm for alpine meadow (AM) under grazing (FG) and grazing exclusion (GE), respectively.**

|  | ANPP | | | Root turnover | | |
| --- | --- | --- | --- | --- | --- | --- |
|  | FG + GE | FG | GE | FG + GE | FG | GE |
|  | 70.45 | 53.00 | 70.45 | 0.313407 | 0.297704 | 0.313407 |
|  | 40.35 | 40.33 | 40.35 | 0.286313 | 0.286299 | 0.286313 |
|  | 54.48 | 36.90 | 54.48 | 0.299036 | 0.28321 | 0.299036 |
|  | 54.12 | 40.04 | 54.12 | 0.298712 | 0.28604 | 0.298712 |
|  | 65.55 | 41.79 | 65.55 | 0.308997 | 0.287613 | 0.308997 |
|  | 53.00 | 61.96 | 89.97 | 0.297704 | 0.305768 | 0.330972 |
|  | 40.33 | 76.89 | 106.79 | 0.286299 | 0.319199 | 0.346109 |
|  | 36.90 | 45.16 | 85.10 | 0.28321 | 0.290644 | 0.326586 |
|  | 40.04 | 53.86 | 70.39 | 0.28604 | 0.298478 | 0.313353 |
|  | 41.79 | 87.13 | 25.15 | 0.287613 | 0.328415 | 0.272637 |
|  | 89.97 | 37.16 | 77.34 | 0.330972 | 0.283444 | 0.31961 |
|  | 106.79 | 31.94 | 59.49 | 0.346109 | 0.27875 | 0.303539 |
|  | 61.96 | 53.86 | 58.74 | 0.305768 | 0.298478 | 0.302866 |
|  | 76.89 | 55.14 | 64.15 | 0.319199 | 0.299626 | 0.307737 |
|  | 85.10 | 69.14 | 46.41 | 0.326586 | 0.312226 | 0.291771 |
|  | 70.39 | 62.43 | 75.78 | 0.313353 | 0.306187 | 0.318202 |
|  | 25.15 | 38.01 | 55.78 | 0.272637 | 0.284211 | 0.300205 |
|  | 45.16 | 76.78 | 98.86 | 0.290644 | 0.319098 | 0.338978 |
|  | 53.86 | 52.30 | 103.84 | 0.298478 | 0.297066 | 0.343456 |
|  | 87.13 | 81.90 | 199.10 | 0.328415 | 0.32371 | 0.429194 |
|  | 77.34 | 118.58 | 98.07 | 0.31961 | 0.356722 | 0.338261 |
|  | 59.49 | 131.29 | 158.39 | 0.303539 | 0.368163 | 0.392553 |
|  | 58.74 | 90.48 | 91.43 | 0.302866 | 0.331432 | 0.332289 |
|  | 37.16 | 52.06 | 56.14 | 0.283444 | 0.29685 | 0.30053 |
|  | 31.94 | 41.34 | 81.77 | 0.27875 | 0.287206 | 0.323595 |
|  | 53.86 | 131.92 | 109.88 | 0.298478 | 0.36873 | 0.348892 |
|  | 64.15 | 21.03 | 18.57 | 0.307737 | 0.268929 | 0.266715 |
|  | 46.41 | 34.81 | 37.23 | 0.291771 | 0.281331 | 0.283505 |
|  | 75.78 | 42.86 | 51.90 | 0.318202 | 0.28857 | 0.296714 |
|  | 55.78 | 35.56 | 61.12 | 0.300205 | 0.282004 | 0.305004 |
|  | 98.86 | 54.68 | 55.82 | 0.338978 | 0.299216 | 0.300242 |
|  | 55.14 | 29.92 | 46.34 | 0.299626 | 0.276924 | 0.291702 |
|  | 69.14 | 45.10 | 59.64 | 0.312226 | 0.290586 | 0.303672 |
|  | 62.43 |  |  | 0.306187 |  |  |
|  | 38.01 |  |  | 0.284211 |  |  |
|  | 76.78 |  |  | 0.319098 |  |  |
|  | 103.84 |  |  | 0.343456 |  |  |
|  | 52.30 |  |  | 0.297066 |  |  |
|  | 199.10 |  |  | 0.429194 |  |  |
|  | 98.07 |  |  | 0.338261 |  |  |
|  | 158.39 |  |  | 0.392553 |  |  |
|  | 81.90 |  |  | 0.32371 |  |  |
|  | 118.58 |  |  | 0.356722 |  |  |
|  | 131.29 |  |  | 0.368163 |  |  |
|  | 91.43 |  |  | 0.332289 |  |  |
|  | 90.48 |  |  | 0.331432 |  |  |
|  | 56.14 |  |  | 0.30053 |  |  |
|  | 81.77 |  |  | 0.323595 |  |  |
|  | 109.88 |  |  | 0.348892 |  |  |
|  | 52.06 |  |  | 0.29685 |  |  |
|  | 41.34 |  |  | 0.287206 |  |  |
|  | 131.92 |  |  | 0.36873 |  |  |
|  | 18.57 |  |  | 0.266715 |  |  |
|  | 37.23 |  |  | 0.283505 |  |  |
|  | 51.90 |  |  | 0.296714 |  |  |
|  | 61.12 |  |  | 0.305004 |  |  |
|  | 21.03 |  |  | 0.268929 |  |  |
|  | 34.81 |  |  | 0.281331 |  |  |
|  | 42.86 |  |  | 0.28857 |  |  |
|  | 35.56 |  |  | 0.282004 |  |  |
|  | 55.82 |  |  | 0.300242 |  |  |
|  | 46.34 |  |  | 0.291702 |  |  |
|  | 59.64 |  |  | 0.303672 |  |  |
|  | 54.68 |  |  | 0.299216 |  |  |
|  | 29.92 |  |  | 0.276924 |  |  |
|  | 45.10 |  |  | 0.290586 |  |  |
| Mean | 65.963 | 58.344 | 73.581 | 0.309 | 0.303 | 0.316 |
| SD | 32.678 | 27.885 | 35.663 | 0.029 | 0.025 | 0.032 |
| SE | 4.022 | 4.854 | 6.208 | 0.004 | 0.004 | 0.006 |

**S1 Table B. Above-ground net primary productivity (ANPP g m^-2^ yr^-1^) and root turnover rates (yr^-1^), which were calculated by Gill’s algorithm for alpine steppe (AS) under grazing (FG) and grazing exclusion (GE), respectively.**

|  | ANPP | | | Root turnover | | |
| --- | --- | --- | --- | --- | --- | --- |
|  | FG + GE | FG | GE | FG + GE | FG | GE |
|  | 23.77 | 18.67 | 23.77 | 0.271391 | 0.266805 | 0.271391 |
|  | 23.81 | 17.24 | 23.81 | 0.271431 | 0.26552 | 0.271431 |
|  | 32.60 | 38.14 | 32.60 | 0.279344 | 0.284326 | 0.279344 |
|  | 40.75 | 19.75 | 40.75 | 0.286677 | 0.267773 | 0.286677 |
|  | 35.95 | 30.55 | 35.95 | 0.282357 | 0.277493 | 0.282357 |
|  | 18.67 | 22.11 | 26.34 | 0.266805 | 0.269901 | 0.273702 |
|  | 17.24 | 40.20 | 24.66 | 0.26552 | 0.28618 | 0.272198 |
|  | 38.14 | 16.68 | 21.86 | 0.284326 | 0.265016 | 0.26967 |
|  | 19.75 | 19.28 | 36.14 | 0.267773 | 0.267356 | 0.282526 |
|  | 30.55 | 18.42 | 26.20 | 0.277493 | 0.266578 | 0.273584 |
|  | 26.34 | 47.55 | 24.14 | 0.273702 | 0.292793 | 0.271726 |
|  | 24.66 | 37.15 | 25.20 | 0.272198 | 0.283433 | 0.27268 |
|  | 21.86 | 17.06 | 41.83 | 0.26967 | 0.265354 | 0.287649 |
|  | 22.11 | 16.73 | 56.23 | 0.269901 | 0.265059 | 0.300609 |
|  | 40.20 | 40.16 | 23.13 | 0.28618 | 0.286144 | 0.270815 |
|  | 16.68 | 29.48 | 27.12 | 0.265016 | 0.276532 | 0.274408 |
|  | 36.14 | 69.41 | 47.07 | 0.282526 | 0.312471 | 0.29236 |
|  | 26.20 | 87.92 | 45.06 | 0.273584 | 0.329132 | 0.290558 |
|  | 24.14 | 50.36 | 42.28 | 0.271726 | 0.29532 | 0.288048 |
|  | 19.28 | 59.16 | 48.58 | 0.267356 | 0.30324 | 0.293722 |
|  | 18.42 | 30.29 | 93.46 | 0.266578 | 0.277263 | 0.334118 |
|  | 47.55 | 15.27 | 66.25 | 0.292793 | 0.263741 | 0.309627 |
|  | 25.20 | 28.61 | 38.92 | 0.27268 | 0.275747 | 0.285028 |
|  | 41.83 | 14.86 | 16.20 | 0.287649 | 0.26337 | 0.26458 |
|  | 56.23 | 15.69 | 17.18 | 0.300609 | 0.264123 | 0.265462 |
|  | 23.13 | 25.71 | 26.56 | 0.270815 | 0.273141 | 0.273908 |
|  | 27.12 | 16.75 | 26.57 | 0.274408 | 0.265073 | 0.273915 |
|  | 37.15 | 16.97 | 24.57 | 0.283433 | 0.265275 | 0.272111 |
|  | 17.06 | 33.82 | 38.30 | 0.265354 | 0.280442 | 0.284474 |
|  | 16.73 | 19.88 | 38.29 | 0.265059 | 0.267888 | 0.284459 |
|  | 40.16 | 50.01 | 32.86 | 0.286144 | 0.295011 | 0.27957 |
|  | 29.48 | 36.14 | 30.15 | 0.276532 | 0.282526 | 0.277137 |
|  | 47.07 | 25.46 | 32.74 | 0.29236 | 0.272918 | 0.279462 |
|  | 69.41 | 33.25 | 43.98 | 0.312471 | 0.279927 | 0.289586 |
|  | 45.06 | 29.63 | 12.64 | 0.290558 | 0.276669 | 0.261372 |
|  | 42.28 | 26.33 | 44.58 | 0.288048 | 0.273699 | 0.290118 |
|  | 48.58 | 19.65 | 30.01 | 0.293722 | 0.267683 | 0.277011 |
|  | 87.92 | 43.51 | 33.48 | 0.329132 | 0.28916 | 0.280132 |
|  | 50.36 | 11.23 | 24.78 | 0.29532 | 0.260105 | 0.272298 |
|  | 59.16 | 19.50 | 11.82 | 0.30324 | 0.26755 | 0.260634 |
|  | 93.46 | 38.98 | 21.37 | 0.334118 | 0.285082 | 0.269235 |
|  | 66.25 | 14.12 | 12.64 | 0.309627 | 0.262704 | 0.261372 |
|  | 38.92 | 7.86 | 15.79 | 0.285028 | 0.25707 | 0.264209 |
|  | 30.29 | 12.85 | 23.45 | 0.277263 | 0.261567 | 0.271107 |
|  | 15.27 | 11.48 | 17.29 | 0.263741 | 0.260328 | 0.265559 |
|  | 28.61 | 9.35 | 16.93 | 0.275747 | 0.258417 | 0.265235 |
|  | 16.20 | 7.86 | 17.86 | 0.26458 | 0.257078 | 0.266078 |
|  | 17.18 | 7.28 | 10.12 | 0.265462 | 0.256548 | 0.259104 |
|  | 26.56 | 9.83 | 25.81 | 0.273908 | 0.258845 | 0.273231 |
|  | 26.57 | 15.08 | 21.10 | 0.273915 | 0.263568 | 0.26899 |
|  | 24.57 | 16.93 | 13.85 | 0.272111 | 0.265239 | 0.262463 |
|  | 14.86 | 13.93 | 16.21 | 0.26337 | 0.262535 | 0.264587 |
|  | 15.69 |  |  | 0.264123 |  |  |
|  | 25.71 |  |  | 0.273141 |  |  |
|  | 16.75 |  |  | 0.265073 |  |  |
|  | 16.97 |  |  | 0.265275 |  |  |
|  | 38.30 |  |  | 0.284474 |  |  |
|  | 38.29 |  |  | 0.284459 |  |  |
|  | 32.86 |  |  | 0.27957 |  |  |
|  | 33.82 |  |  | 0.280442 |  |  |
|  | 19.88 |  |  | 0.267888 |  |  |
|  | 50.01 |  |  | 0.295011 |  |  |
|  | 30.15 |  |  | 0.277137 |  |  |
|  | 32.74 |  |  | 0.279462 |  |  |
|  | 43.98 |  |  | 0.289586 |  |  |
|  | 36.14 |  |  | 0.282526 |  |  |
|  | 25.46 |  |  | 0.272918 |  |  |
|  | 33.25 |  |  | 0.279927 |  |  |
|  | 12.64 |  |  | 0.261372 |  |  |
|  | 44.58 |  |  | 0.290118 |  |  |
|  | 30.01 |  |  | 0.277011 |  |  |
|  | 29.63 |  |  | 0.276669 |  |  |
|  | 26.33 |  |  | 0.273699 |  |  |
|  | 19.65 |  |  | 0.267683 |  |  |
|  | 33.48 |  |  | 0.280132 |  |  |
|  | 43.51 |  |  | 0.28916 |  |  |
|  | 24.78 |  |  | 0.272298 |  |  |
|  | 11.82 |  |  | 0.260634 |  |  |
|  | 21.37 |  |  | 0.269235 |  |  |
|  | 11.23 |  |  | 0.260105 |  |  |
|  | 19.50 |  |  | 0.26755 |  |  |
|  | 38.98 |  |  | 0.285082 |  |  |
|  | 12.64 |  |  | 0.261372 |  |  |
|  | 15.79 |  |  | 0.264209 |  |  |
|  | 23.45 |  |  | 0.271107 |  |  |
|  | 14.12 |  |  | 0.262704 |  |  |
|  | 7.86 |  |  | 0.25707 |  |  |
|  | 12.85 |  |  | 0.261567 |  |  |
|  | 17.29 |  |  | 0.265559 |  |  |
|  | 16.93 |  |  | 0.265235 |  |  |
|  | 17.86 |  |  | 0.266078 |  |  |
|  | 10.12 |  |  | 0.259104 |  |  |
|  | 25.81 |  |  | 0.273231 |  |  |
|  | 11.48 |  |  | 0.260328 |  |  |
|  | 9.35 |  |  | 0.258417 |  |  |
|  | 7.86 |  |  | 0.257078 |  |  |
|  | 7.28 |  |  | 0.256548 |  |  |
|  | 9.83 |  |  | 0.258845 |  |  |
|  | 21.10 |  |  | 0.26899 |  |  |
|  | 13.85 |  |  | 0.262463 |  |  |
|  | 16.21 |  |  | 0.264587 |  |  |
|  | 15.08 |  |  | 0.263568 |  |  |
|  | 16.93 |  |  | 0.265239 |  |  |
|  | 13.93 |  |  | 0.262535 |  |  |
| Mean | 28.294 | 26.426 | 30.163 | 0.275 | 0.274 | 0.277 |
| SD | 15.737 | 16.384 | 14.987 | 0.014 | 0.015 | 0.013 |
| SE | 1.543 | 2.272 | 2.078 | 0.001 | 0.002 | 0.002 |

**S1 Table C. Above-ground net primary productivity (ANPP g m^-2^ yr^-1^) and root turnover rates (yr^-1^), which were calculated by Gill’s algorithm for alpine desert steppe (ADS) under grazing (FG) and grazing exclusion (GE), respectively.**

|  | ANPP | | | Root turnover | | |
| --- | --- | --- | --- | --- | --- | --- |
|  | FG + GE | FG | GE | FG + GE | FG | GE |
|  | 9.42 | 10.62 | 9.42 | 0.258482 | 0.259562 | 0.258482 |
|  | 10.92 | 12.82 | 10.92 | 0.259832 | 0.261538 | 0.259832 |
|  | 14.42 | 15.78 | 14.42 | 0.262978 | 0.264198 | 0.262978 |
|  | 9.09 | 13.89 | 9.09 | 0.258179 | 0.262499 | 0.258179 |
|  | 8.52 | 13.98 | 8.52 | 0.257672 | 0.262578 | 0.257672 |
|  | 10.62 | 23.47 | 7.84 | 0.259562 | 0.271121 | 0.25706 |
|  | 12.82 | 13.61 | 15.04 | 0.261538 | 0.262251 | 0.263536 |
|  | 15.78 | 21.78 | 12.20 | 0.264198 | 0.269598 | 0.26098 |
|  | 13.89 | 17.14 | 22.80 | 0.262499 | 0.265426 | 0.270516 |
|  | 13.98 | 19.02 | 27.94 | 0.262578 | 0.267122 | 0.275142 |
|  | 7.84 | 7.63 | 12.84 | 0.25706 | 0.256869 | 0.261552 |
|  | 15.04 | 9.23 | 12.16 | 0.263536 | 0.258309 | 0.26094 |
|  | 23.47 | 10.29 | 6.22 | 0.271121 | 0.259263 | 0.255602 |
|  | 13.61 | 8.13 | 4.86 | 0.262251 | 0.257319 | 0.254378 |
|  | 12.20 | 6.44 | 10.16 | 0.26098 | 0.2558 | 0.259148 |
|  | 22.80 | 5.54 | 6.34 | 0.270516 | 0.254982 | 0.255702 |
|  | 27.94 | 9.59 | 7.75 | 0.275142 | 0.258633 | 0.256977 |
|  | 21.78 | 6.49 | 7.87 | 0.269598 | 0.255839 | 0.257085 |
|  | 17.14 | 32.44 | 21.88 | 0.265426 | 0.279192 | 0.269692 |
|  | 19.02 | 24.25 | 22.57 | 0.267122 | 0.271823 | 0.270315 |
|  | 12.84 | 16.45 | 21.46 | 0.261552 | 0.264807 | 0.26931 |
|  | 12.16 |  |  | 0.26094 |  |  |
|  | 6.22 |  |  | 0.255602 |  |  |
|  | 7.63 |  |  | 0.256869 |  |  |
|  | 9.23 |  |  | 0.258309 |  |  |
|  | 10.29 |  |  | 0.259263 |  |  |
|  | 4.86 |  |  | 0.254378 |  |  |
|  | 10.16 |  |  | 0.259148 |  |  |
|  | 6.34 |  |  | 0.255702 |  |  |
|  | 7.75 |  |  | 0.256977 |  |  |
|  | 7.87 |  |  | 0.257085 |  |  |
|  | 8.13 |  |  | 0.257319 |  |  |
|  | 6.44 |  |  | 0.2558 |  |  |
|  | 5.54 |  |  | 0.254982 |  |  |
|  | 9.59 |  |  | 0.258633 |  |  |
|  | 6.49 |  |  | 0.255839 |  |  |
|  | 21.88 |  |  | 0.269692 |  |  |
|  | 22.57 |  |  | 0.270315 |  |  |
|  | 21.46 |  |  | 0.26931 |  |  |
|  | 32.44 |  |  | 0.279192 |  |  |
|  | 24.25 |  |  | 0.271823 |  |  |
|  | 16.45 |  |  | 0.264807 |  |  |
| Mean | 13.593 | 14.218 | 12.967 | 0.262 | 0.263 | 0.262 |
| SD | 6.717 | 6.954 | 6.580 | 0.006 | 0.006 | 0.006 |
| SE | 1.036 | 1.518 | 1.436 | 0.001 | 0.001 | 0.001 |
